# An Efficient Turbo Decoding and Frequency Domain Turbo Equalization for LTE Based Narrowband Internet of Things (NB-IoT) Systems

**DOI:** 10.3390/s21165351

**Published:** 2021-08-08

**Authors:** Mohammed Jajere Adamu, Li Qiang, Rabiu Sale Zakariyya, Charles Okanda Nyatega, Halima Bello Kawuwa, Ayesha Younis

**Affiliations:** 1 School of Microelectronics, Tianjin University, Tianjin 300072, China; liqiang@tju.edu.cn (L.Q.); ayesha@tju.edu.cn (A.Y.); 2 Department of Electronics Science and Technology, University of Science and Technology of China (USTC), Hefei 230026, China; rabiu123@mail.ustc.edu.cn; 3 School of Electrical and Information Engineering, Tianjin University, Tianjin 300072, China; ncharlz@tju.edu.cn; 4 School of Precision Instrument and Opto-Electronics Engineering, Tianjin University, Tianjin 300072, China; halima@tju.edu.cn

**Keywords:** Narrowband IoT (NB-IoT), narrowband physical uplink shared channel (NPUSCH), bit error rate (BER), maximum a posteriori probability (MAP), minimum mean square error (MMSE)

## Abstract

This paper addresses the main crucial aspects of physical (PHY) layer channel coding in uplink NB-IoT systems. In uplink NB-IoT systems, various channel coding algorithms are deployed due to the nature of the adopted Long-Term Evolution (LTE) channel coding which presents a great challenge at the expense of high decoding complexity, power consumption, error floor phenomena, while experiencing performance degradation for short block lengths. For this reason, such a design considerably increases the overall system complexity, which is difficult to implement. Therefore, the existing LTE turbo codes are not recommended in NB-IoT systems and, hence, new channel coding algorithms need to be employed for LPWA specifications. First, LTE-based turbo decoding and frequency-domain turbo equalization algorithms are proposed, modifying the simplified maximum a posteriori probability (MAP) decoder and minimum mean square error (MMSE) Turbo equalization algorithms were appended to different Narrowband Physical Uplink Shared Channel (NPUSCH) subcarriers for interference cancellation. These proposed methods aim to minimize the complexity of realizing the traditional MAP turbo decoder and MMSE estimators in the newly NB-IoT PHY layer features. We compare the system performance in terms of block error rate (BLER) and computational complexity.

## 1. Introduction

Internet of things (IoT) systems have been one of the fast-growing communications technologies in recent years and enable the massive connectivity of consumer electronic devices including smart sensors, actuators, Bluetooth, radio frequency identification (RFID) tags, ZigBee, and smart devices that are accessible to the internet for transforming ideas and working systems. It is expected that in the near future, massive number of devices within distributed objects will be connected to the wireless network through a common addressing scheme, which brings in the evolution of new intelligent service systems available around the globe. Besides, different existing and emerging wireless communication technologies are facing a lot of challenges in realizing the objective of loT communications, including enhanced network coverage, low data rates, high complexity, and long delay sensitivity [1].

**Figure 1:**
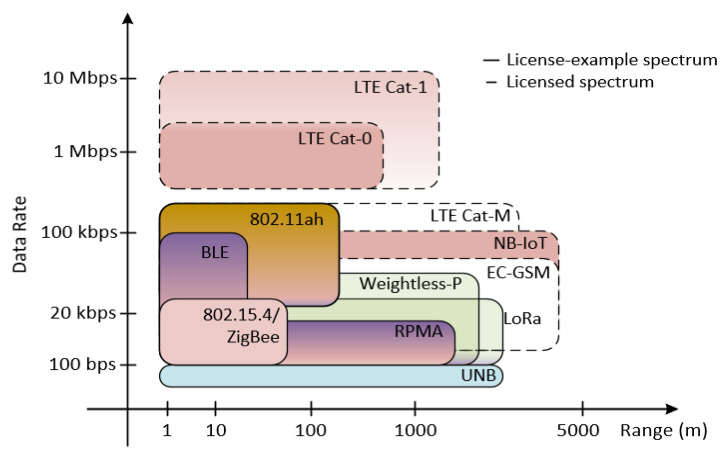
Classification of LPWAN networks based on data rate and signal range.

Most of the delay in transmission s from low throughput and cost-effective communication that provides the connected IoT to the globe. The enormous communication required for various low data rate IoT devices designed to provide new services beyond the Device to Device (D2D) and Human to Human (H2H) protocols regarding transmission reliability and delay sensitivity, cannot be adopted by the existing cellular communication standard efficiently. These devices are conceived for a different class of point to point communication that operates with a high power consumption and possesses high design complexity leading to implementation cost and deployment difficulty [2]. Beside this, protocols such as ZigBee, Wi-Fi and Bluetooth proffer short coverage communication and demand low power consumption, but if deployed continually would cost more. Therefore, this is not a cost-effective solution for applications with wide coverage requirements such as MIoT devices that need to support large-scale connections with low power consumption at reduced cost, and provide wide coverage over LTE and GSM networks as illustrated in Figure 1.

In recent years, different IoT standardization bodies have proposed different technologies to accommodate the requirements of IoT applications in current and future cellular networks. Specifically, a novel cellular-based low power wide-area (LPWA) IoT technology, known as the narrowband loT (NB-loT) system, has been standardized by the third Generation Partnership Project (3GPP), where the most recent release is dedicated to serve some part of fifth-generation (5G) technology [3]. Table 1 below, presents the technical specification of LPWA network technology. To improve the NB-IoT LPWA specification, release-14 of the 3GPP is supplemented with features relevant for mMTC use case where device positioning/localization, as well as cell search improvement are introduced, which focuses on high peak data rates with UE power allocation of 23 dBm and enhanced cell ID [4]. Release-14 integrates NB-IoT into 5G in addition to low latency reduction, new wake-up receiver design for higher density support, improved operation, and multiple Hybrid Automatic Repeat Request (HARQ) feedback, which realizes better throughput with repetitive transmission. In NB-IoT, multiple associated control signaling with repeating transmission data have been utilized as the main solution to achieve extended network coverage for NB-IoT systems. Other literature on NB-IoT takes into consideration the new feature of repetition. Besides that, some methods consider performing a link adaptation for resource management to enhance data rate and power reduction. In addition, coverage efficiency through link adaptation, and modulation and coding scheme (MCS) index selection are considered in existing LTE technology, as well as the determination of the appropriate number of repetitions [5,6,7,8,9,10,11,12].

Wireless IoT systems are expected to offer coverage reachability of about 164 dB, battery life of 10 years, coverage density to support up to 10 million devices in a square km^2^, and a system throughput of 200 bps with processing latencies lower than 10 s [13,14]. Hence, choosing a suitable PHY layer technology is essential to meet the requirement. However, as specified in the 3GPP standard, NB-IoT is designed based on existing LTE PHY layer technology, with its baseband signals and channels inherited from LTE with additional repetition parameters to meet the demand. In the uplink technology, NB-IoT adopted LTE turbo coding as FEC and SC-FDMA as the multiplexing scheme [15,16] due to its advantage of Peak-to-Average Power Ratio (PAPR) that results from efficient power amplification compared to ordinary OFDMA technology. The main goal of Forward Error Correction (FEC) is to improve the transmission reliability with which user data may be conveyed through a noisy channel, such as a wireless communication channel or an imperfect information system. This is achieved by the addition of redundant information derived from the data to be conveyed prior to transmission over the noisy channel [17]. At the destination, the redundant bit of the transmitted data may be exploited in multiplex with the original information message to correct errors that may have been introduced by the channel [18].

Turbo codes for modern error correction coding provide high error rate performance near the Shannon limits of error-correcting performance [19]. These excellent features of turbo codes have led to their adoption in many communication standards, including high-speed downlink packet access (HSDPA), wireless local area network (WLAN), LTE/LTE-A, and some portion of mMTC in 5G technology, although the performance of turbo codes comes at the expense of high decoding complexity and consequently high-power consumption [16] at the receiver for proper decoding. Actually, the computational difficulty of the turbo decoder far exceeds that of other components in an LTE and NB-IoT receiver, especially for higher data rate and low latency communications. To make the uplink system more flexible, a frequency-domain minimum mean squared error (MMSE) equalizer in a soft interference cancellation (SIC) scheme has been used in the LTE uplink receiver for SC-FDMA. Motivated by this, the frequency domain equalizer (FDE) can be utilized [16]. Several iterative turbo decoding and equalization receiver schemes have been proposed in the literature [20,21]. In [21], during the iterations, the equalizer coefficients remained constant, while in [22] during the iterations, the equalizer was updated in conformity with a priori information received from the decoder. It was presented in [23] that frequency domain equalization (FDE) can easily be applied to uplink NB-IoT system to eliminate the Inter-Symbol Interference (ISI) effect.

To further resolve this problem, efficient turbo decoding and equalization is needed, where the frequency domain equalizer and the turbo channel decoder can jointly exchange their soft-decision information by performing iterative detection, thereby canceling the ISI. The turbo decoding and FD-TE considered in the receiver are first formulated to estimate the ISI and sensitivity error among the NPUSCHs subcarrier. By using the repeated data sequence and symbols selection on the NPUSCH subcarriers, the residual ISI can be estimated. Then, the FD-TE equalizer can be introduced using the minimum mean square (MMSE) [22] estimation method and circulant matrix operations to compute the frequency domain estimates of the received signal. The proposed receiver jointly performs the equalization and turbo decoding on specific NPUSCH resources. The analytical results are verified by the mean square error (MSE) and block error rate (BLER) simulation performance of the proposed NPUSCH receiver. Finally, this paper presents a sufficiently robust scheme compared to the conventional FDTE MMSE, which can improve the reliability of the transmission of UE data in NPUSCH and thus reduce the computational complexity suitable for a LPWA NBIoT system.

The rest of this paper is organized as follows: Our proposed system model is described in Section 2. The numerical results and complexity analysis are presented in Section 3. The conclusion and future work recommendations are presented in Section 4.

## 2. System Model

**Figure 2:**
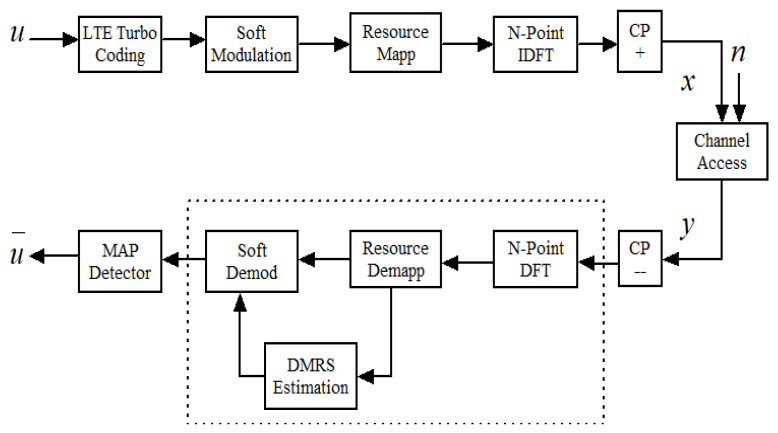
System model of LTE based uplink NB-IoT systems.

Consider the uplink PHY layer data transmission shared channel of NB-IoT system with k=1,…,K, UE of $u_{k}=\left(u_{0},u_{1},\ldots ,u_{m-1}\right),u_{i}\in \left\{0,1\right\}$ transport blocks (TBs), which are simultaneously transmitted on their NPUSCH to the base station (BS) for network access as illustrated in Figure 2. The model employs a single-tone SC-FDMA with $N_{k}$ contiguous subcarriers spaced with $\Delta f_{n}=15\;\mathrm{kHz}$ and $\Delta f_{n}=$ 3.75 kHz SCS, respectively. Since the transmission bandwidth is limited to 180 kHz, the NPUSCH frame in the time domain consists of a pair of slots each consisting of 7OFDM symbols with one NDMRS as a pilot-aided symbol. For $\Delta f_{n}=15\;\mathrm{kHz}$ SCS, the resource grid composed of 12 subcarriers per 0.5 ms slot duration forms one physical resource block (PRB). Similarly, for $\Delta f_{n}=3.75\;\mathrm{kHz}$ SCS, 48 subcarriers are used per 2 ms slot duration forming one PRB.

A u TB to be transported on NPUSCH is encoded through the LTE Turbo encoder. Recursive Systematic Convolutional (RSC) codes are primarily applied as the constituent component codes. At any time, k, the input to the encoder is a bit $u_{k}$, which is converted to the corresponding code bit $c_{k}$ based on the generator polynomials defined by

(1) $$G\left(D\right)=\left[1,\frac{g_{1}\left(D\right)}{g_{0}\left(D\right)}\right]$$

where $g_{1}=1+D^{2}+D^{3}$ and $g_{0}=1+D+D^{3}$ and each is separated internally by interleaver π at 1/3 coding rate (R) resulting in an encoded sequence which is rate-matched and mapped onto a constellation of binary phase-shift keying (BPSK). In other words, mapping from {0,1} to {−1,+1} with the signal constellation of w=2x−1 modulates signals and transmits over the wireless channel. The coded symbols generate four redundancy versions (RV), which are selected among the sequence {0,2,3,1} of the TB as code blocks [24]. Each RV is transmitted in one subframe available in each NPUSCH, which is configured into RU to schedule for transmission. The resultant code bits are mapped on $a_{k}$ symbols. The resulting samples are transformed to time domain through the $N_{k}$ Inverse Discrete Fourier Transform (IDFT) along the n-the subcarrier as follows:

(2) $$x_{k}=\frac{1}{\sqrt{N_{k}}}\sum_{n=0}^{N_{k}-1}a_{k}e^{j2\pi n\Delta f_{n}},0\leq n\leq N_{k}-1$$

where $\Delta f_{n}$ is the subcarrier spacing (which can either be 15 kHz or 3.75 kHz).

To block inter-block interference (IBI), a cyclic prefix (CP) is appended to the $x_{n}$, then the samples are transmitted simultaneously over a wireless channel. Assuming perfect synchronization at the receiver, the CP is discarded, the N-point DFT is applied, then the discrete-time domain equivalent baseband received signals can be expressed as follows

(3) $$y_{k}=\sum_{k=1}^{K-1}H_{k}x_{k}+z$$

where $H_{k}$ denotes the CIR matrix with ${\left[h_{k,0},h_{k,1},\ldots ,h_{k,L-1},0,\ldots ,0\right]}^{T}$ as its column entry and z is the additive white Gaussian noise (AWGN) vector having a zero-mean and variance of $\sigma_{z}^{2}$. After receiving the $y_{k}$ symbol, maximum a posterior probability (MAP) detection is performed to compute the likelihood for every demodulated symbol to deliver a soft symbol for decoding. The process is repeated for certain iterations to obtain a desired decoded output bit.

### 2.1. MAP Turbo Decoding

**Figure 3:**
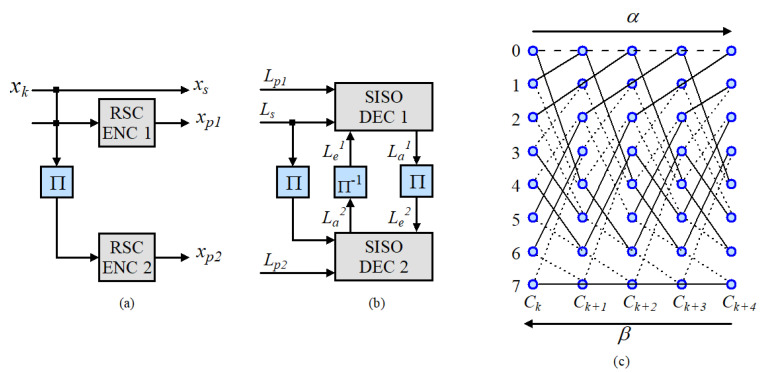
Block diagram of (**a**) turbo encoder, (**b**) decoder, and the (**c**) state transition diagram of turbo codes.

As shown in Figure 3, the turbo decoder performs decoding processes by exploiting a maximum a posteriori (MAP) algorithm, where the received signal sequence is demultiplexed into three sequences [25]: the systematic sequence $y^{s}$ and two parity sequences $y^{p1}$ and $y^{p2}$. One SISO decoder utilizes $y^{s}$ and $y^{p1}$ (or $y^{p2}$) as inputs and computes the log-likelihood ratio (LLR) of each information bit based on the trellis structure in Figure 3c, which is defined for the k-th information bit $u_{k}$, as

(4) $$\begin{array}{c} L\left({\hat{u}}_{k}\right)=\log\frac{P\left(u_{k}=+1|y\right)}{P\left(u_{k}=-1|y\right)}\;\;\;\;\;\\ \;\;\;\;=\log(\frac{\sum_{u_{k}=+1}{\tilde{\alpha }}_{k\sum 1}(s^{\prime }){\tilde{\beta }}_{k}(s){\bar{\gamma }}_{k}\left(s^{\prime },s\right)}{\sum_{u_{k}=\sum 1}{\tilde{\alpha }}_{k\sum 1}(s^{\prime }){\tilde{\beta }}_{k}(s){\tilde{\gamma }}_{k}\left(s^{\prime },s\right)}) \end{array}$$

where ${\tilde{\alpha }}_{k}\left(s\right),{\overset{¯}{\beta }}_{k}\left(s\right),$ and ${\tilde{\gamma }}_{k}\left(s^{\prime },s\right)$ denote the forward, backward and branch metrics, respectively. The s and $s^{\prime }$ indexes are associated with trellis steps k and k−1, respectively. The MAP algorithm traverses in both the forward and backward directions to obtain state metrics ${\tilde{\alpha }}_{k}\left(s\right)$ and $\beta_{k}\left(s\right),$ respectively. In the k-th stage, the transmission value from the $s^{\prime }$ state to the s state is denoted by ${\tilde{\gamma }}_{k}\left(s^{\prime },s\right).$ The ${\tilde{\alpha }}_{k}\left(s\right){\tilde{\beta }}_{k}\left(s\right),$ and ${\tilde{\gamma }}_{k}\left(s^{\prime },s\right)$ matrices are computed as

(5) $${\tilde{\alpha }}_{k}(s)=\sum_{s^{\prime }}{\tilde{\gamma }}_{k}\left(s^{\prime },s\right){\tilde{\alpha }}_{k-1}\left(s^{\prime }\right)$$

(6) $${\tilde{\beta }}_{k-1}\left(s^{\prime }\right)=\sum_{s}{\overset{¯}{\gamma }}_{k}\left(s^{\prime },s\right){\overset{¯}{\beta }}_{k}(s)$$

(7) $${\tilde{\gamma }}_{k}\left(s^{\prime },s\right)=\exp\left[\frac{1}{2}L_{e}\left(u_{k}\right)u_{k}+\frac{1}{2}L_{c}X_{k}^{s}u_{k}+\frac{1}{2}L_{c}X_{k}^{p}c_{k}\right]$$

where $L_{e}\left(u_{k}\right)$ denotes the extrinsic LLR value of $u_{k},L_{c}$ is the channel reliability measure and $X_{k}^{s}$ and $X_{k}^{p}$ are the transmitted bits of $x_{k}^{S}$ and $x_{k}^{p},$ respectively. Due to the high computational complexity of the MAP algorithm, which is a result of exponential and multiplication calculations, usually, an equivalent logarithmic form is applied, where multiplication is changed to an addition. Therefore, the equivalent equations in (5)–(7) can be rewritten in the following [14] matrices computed as

(8) $$\alpha_{k}(s)=\log\left[\sum_{s^{\prime }}\exp\left(\gamma_{k}^{\prime }\left(s^{\prime },s\right)+\alpha_{k-1}\left(s^{\prime }\right)\right)\right]$$

(9) $$\beta_{k-1}\left(s^{\prime }\right)=\log\left[\sum_{s}\exp\left(\gamma_{k}^{\prime }\left(s^{\prime },s\right)+\beta_{k}(s)\right)\right]$$

(10) $$\gamma_{k}^{\prime }\left(s^{\prime },s\right)=\frac{1}{2}L_{e}\left(u_{k}\right)u_{k}+\frac{1}{2}L_{c}X_{k}^{s}u_{k}+\frac{1}{2}L_{c}X_{k}^{p}c_{k}$$

where the quantities ${\overset{¯}{\alpha }}_{k}\left(s\right),{\overset{¯}{\beta }}_{k}\left(s\right),$ and ${\overset{¯}{\gamma }}_{k}\left(s^{\prime },s\right)$ are defined in the following [26]

(11) $$\alpha_{k}\left(s\right)=\log\left({\tilde{\alpha }}_{k}\left(s\right)\right)$$

(12) $$\beta_{k}\left(s\right)=\log\left({\tilde{\beta }}_{k}\left(s\right)\right)$$

(13) $$\gamma_{k}^{\prime }\left(s^{\prime },s\right)=\log\left({\tilde{\gamma }}_{k}\left(s^{\prime },s\right)\right)$$

The above-stated logarithmic computation of the SISO MAP decoding algorithm is utilized to make the hardware implementation of the algorithm realizable. The $\gamma^{\prime }$ values are easily achieved through a few numbers of additions that are stable in hardware. The computation of α,β, and LLR values constitute the major computational part of the algorithm, which has a high impact on power consumption and silicon area.

### 2.2. Frequency Domain Turbo Equalization

The FD-TE is involved in turbo decoding and equalization processes of the $y_{k,s}$ symbols, where residual ISI is canceled by applying the MMSE criterion [22] iteratively and improves the error rate performance. After IDFT is performed at the FDTE equalizer output, the time-domain estimate of the X(n) vector is obtained as

(14) $$\hat{X}\left(n\right)=W{\left(n\right)}^{H}Y+C\left(n\right)$$

where W(n) is the weight vector and C(n) is the equalizer multiplicative parameter. Then, following the MMSE criterion, W(n) vector is obtained by

(15) $$w_{n}={\left[\sigma_{w}^{2}I_{N}+H_{eq}VH_{eq}^{H}\right]}^{-1}H_{eq}v_{k}e_{n}$$

where V=diag[v(0),v(1),…,v(N−1)] denotes the diagonal matrix of the estimated symbols, $I_{N}$ is an N×N identity matrix, and $e_{n}={\left[0_{1\times n}10_{1\times \left(N-n-1\right)}\right]}^{T}\;$ is the n-th length unit vector, following the extrinsic LLR $L_{c}\left(u\left(j\right)\right)$ of the MAP detector in (1) and applying the MMSE criterion to the FDTE output by minimizing the MSE $\left\{|x\left(n\right)-\hat{x}\left(n\right){|}^{2}\right\}$. Then, from the a priori LLRs ${\left[L_{\varepsilon }\left(u\left(j\right)\right)\right]}_{j-0}^{2N-1}$ of the MAP detector in (4), the mean ${\overset{¯}{x}}_{k}$ and the corresponding variance $v_{k}^{1}$ of the hard symbol estimates $x_{k}$ are computed as [16]

(16) $$\begin{array}{c} \mu_{x,k}=\frac{1}{\sqrt{2}}\left[\tan h\left(\frac{\lambda_{2m}^{e}}{2}\right)+j\tan h\left(\frac{\lambda_{2}^{c}m+1}{2}\right)\right]\\ v_{k}=1-{\left|\mu_{z,k}\right|}^{2} \end{array}$$

Here, we consider the particular case of the minimization of the mean-square error (MSE) by minimizing the MSEE $\left\{|x\left(n\right)-\hat{x}\left(n\right){|}^{2}\right\}$. Based on Gaussian distribution, the generated extrinsic LLR ${\left[L_{c}\left(u\left(j\right)\right)\right]}_{j-0}^{2N-1}$ of the SISO decoder is the real and imaginary part of the n-th given by

(17) $$\lambda_{2m}^{e}=\frac{\sqrt{8}Re\left\{\hat{s}\left(n\right)\right\}}{1-v\mu_{\dot{s}}}$$

and

(18) $$\lambda_{2m+1}^{e}=\frac{\sqrt{8}Im\left\{s\left(n\right)\right\}}{1-v\mu_{\dot{s}}}$$

where $\hat{s}\left(n\right)=g_{n}^{H}r+c\left(n\right)$ is the frequency domain estimate. To avoid complexity in computing the V matrix from (15), a truncating method is employed to approximate V by $vI_{N}=\mathrm{trace}\left(V\right)I_{N}$ (as in [20]). By applying the one-tap MMSE criterion and the knowledge of the a priori LLR provided by decoder form (17)–(18), the estimated vector ${\hat{x}}_{n,k}$ can be expressed as

(19) $${\hat{X}}_{0}=\left(Y-xH_{eq}\right)C_{0}^{H}+g_{0}^{H}H_{eq}e_{0}\bar{x}$$

where $C_{0}$ is an estimated frequency-domain circulant matrix with go as the first column. If we define $A≜\sigma^{2}I_{N\times N}+$ $vC_{h}C_{h}^{H}$ and apply the matrix inversion lemma to (1), we have

(20) $$\hat{S}={\left[G_{0}\right]}^{H}Y-\left[{\left[G_{0}\right]}^{H}\Lambda^{eq}-\mu_{s,k}I_{N}\right]\bar{S}$$

where $G_{0}=Fg_{0}$ is the frequency domain version of weight vector and $\Lambda^{eq}=FH^{eq}F^{H}$ is a diagonal matrix whose (k,k)-th entry $\Lambda^{-q}\left(k\right)$ is equal to the *k*-th DFI coefficient, and $\mu_{s,k}=\frac{1}{N}\sum_{k=0}^{N-1}\left(G_{0}^{*}(k){ℋ}_{\mu ,e}(n)\right)$ are complementary equalizer parameters for ISI cancellation. From (20), the frequency domain equalizer coefficient $G_{0}\left(k\right)$ and estimate $\hat{S}\left(k\right)$ at the k-th subcarrier can be expressed as [22]

(21) $$\begin{array}{c} G_{0}\left(k\right)=\frac{{ℋ}_{\mu ,e}\left(n\right)}{\sigma_{w}^{2}+v{\left|{ℋ}_{\mu ,e}\left(n\right)\right|}^{2}}\\ \hat{S}\left(k\right)=G_{0}^{*}\left(k\right)Y\left(k\right)-\left[G_{0}^{*}\left(k\right){ℋ}_{\mu ,e}\left(n\right)-\mu_{s}\right]\overset{¯}{S}\left(k\right) \end{array}$$

As presented in (21), the filter coefficients change the feedback information in different iterations. It is evident from (15), the matrix inversion is carried out on small matrix of size Q×Q, and equalization performed on each NPUSCH SCS.

## 3. Numerical Results and Complexity Analysis

Here, we present a computer simulation to verify the performance of the proposed NPUSCH receiver scheme in terms of bit error rate and throughput under a specific SNR. In addition, the complexity of the entire system is analyzed in detail.

### 3.1. Simulation Setup

The main 3GPP LTE-based PHY layer transmission of NB-IoT systems in MATLAB [27] was used for the link level simulation, and the bandwidth of the NBIoT system was selected as 180 kHz and 900 MHz carrier bandwidth respectively, i.e., single-tone or multi-tone transmission. Multi-tone transmission is an optional numerology that uses the same 15 kHz LTE SCS with 3, 6, 12 as the acceptable number of tones. However, for single tone transmission, 3.75 kHz and 15 kHz are satisfactory SCS options. For multiple tone transmission, RU programming assigns 1 ms for 12 tones, 2 ms for 6 tones, 4 ms for 3 tones, 8 ms for individual tones, 4 ms for 3 ms, 2 ms for 6 tones and 1 ms for 12 tones, respectively. Note that 15 kHz SCS is similar to LTE and provides better performance. When single tone transmission is used, a new radio frame structure is defined from a time domain perspective.

We considered the uplink single-tone NPUSCH transmission with $\Delta f_{n}=15\;\mathrm{kHz}$ and $\Delta f_{n}=3.75\;\mathrm{kHz}$ with $N_{s}=1.92\;\mathrm{MHz}$ as the sampling rate, M=128 as DFT size and $N_{cp}=35$. The channel imposed six taps over wireless multipath fading plus AWGN. For channel coding, the LTE Turbo code with code rates of 1/2 and 1/3 were used and the equalizer was implemented using a soft-output MAP algorithm. However, MAP equalization was implemented by the BCJR algorithm with the number of ISI channel taps L=6. A TBs length of 1000 bits (corresponds to $N_{RU}=4$ at MCS=12 in Table 2.2 of [28] was scheduled on NPUSCH which was mapped to QPSK constellation using Gray coding. The proposed ML-based channel estimation technique was performed using two NDMRS symbols per subframe whose parameter was set to ϵ=0.211.

### 3.2. BER Performance

**Figure 4:**
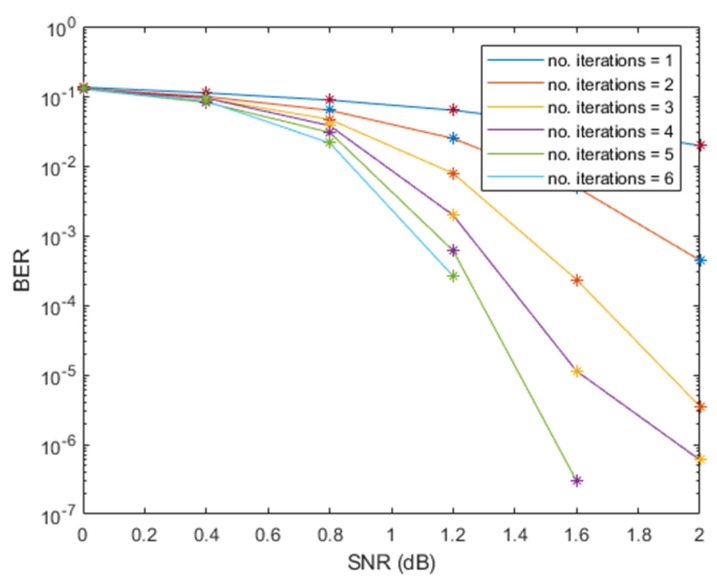
BER performance of Turbo decoding using $\Delta f_{n}=15\;\mathrm{kHz}$.

**Figure 5:**
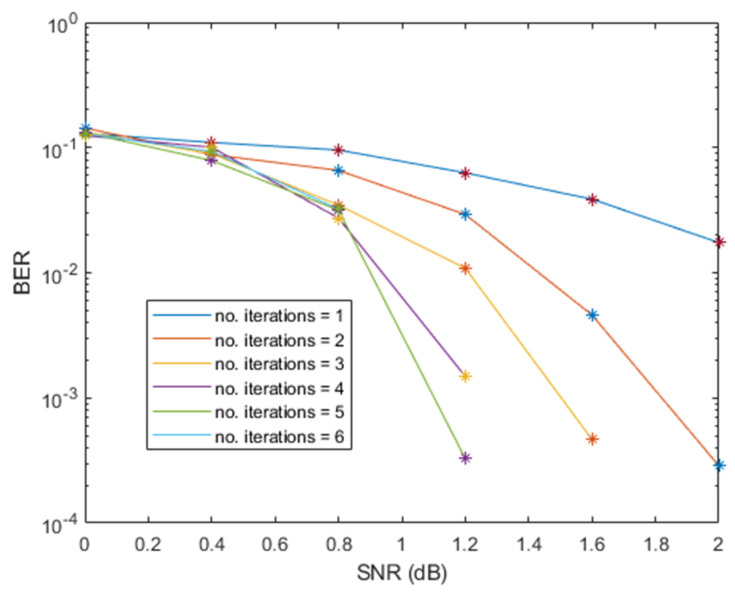
BER performance of Turbo decoding using $\Delta f_{n}=3.75\;\mathrm{kHz}$.

We evaluated the bit error rate (BER) performance for the number of decoding iterations against signal-to-noise ratio (SNR) as shown in Figure 4 and Figure 5, respectively. The BER performance of this scheme was achieved after I = 7 iterations were performed.

**Figure 6:**
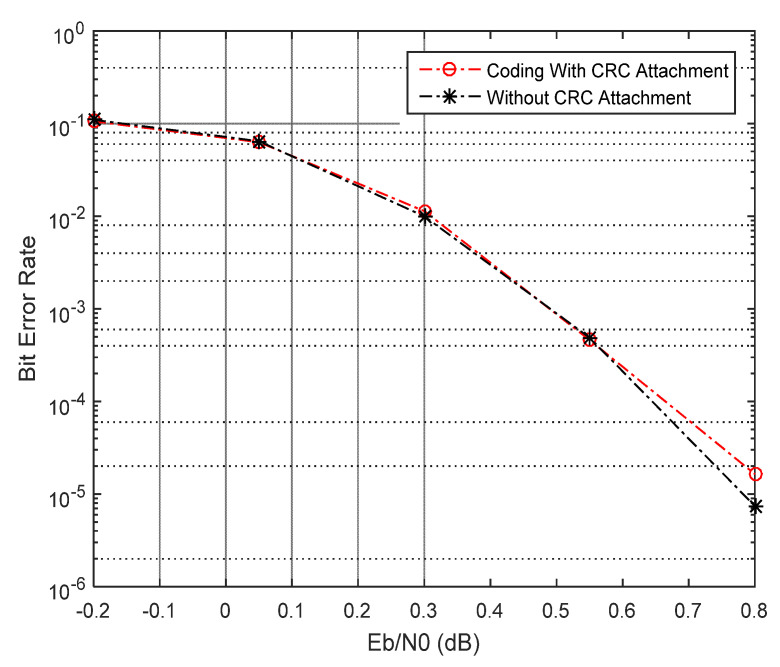
BER performance with CRC mechanism.

**Figure 7:**
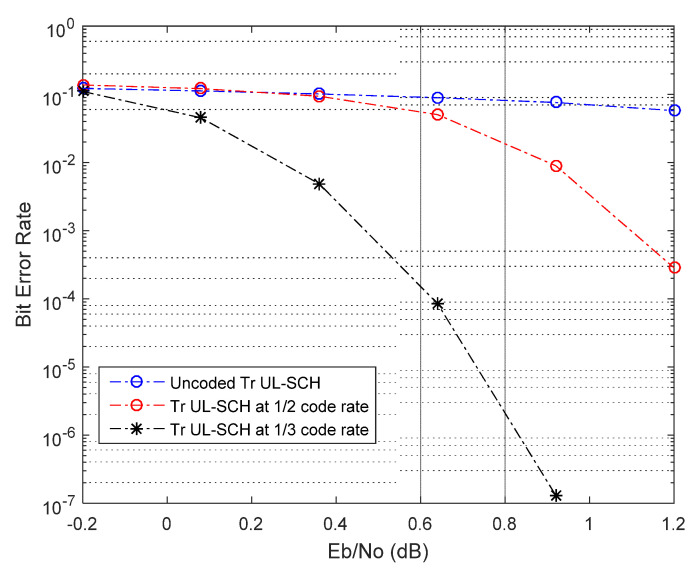
BER performance of Turbo decoding with different code rates.

**Figure 8:**
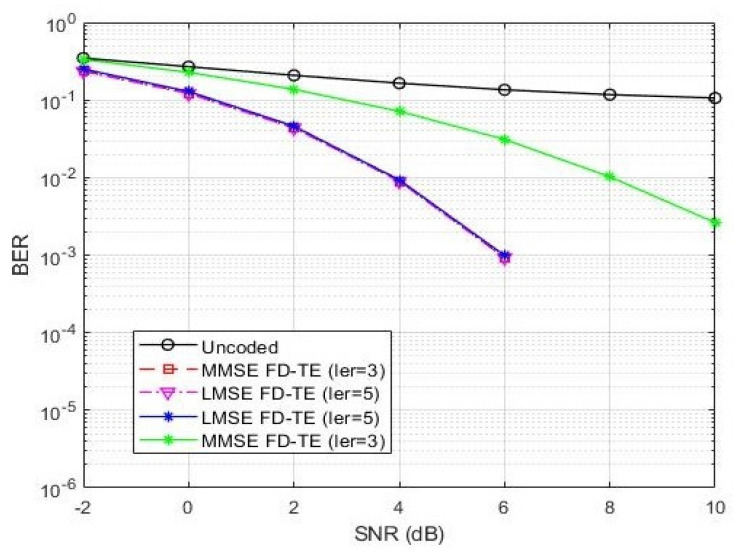
BER performance comparison of the proposed MMSE-FDTE and LMSE equalization algorithms of uplink NB-IoT systems using BPSK with different numbers of iterations.

**Figure 9:**
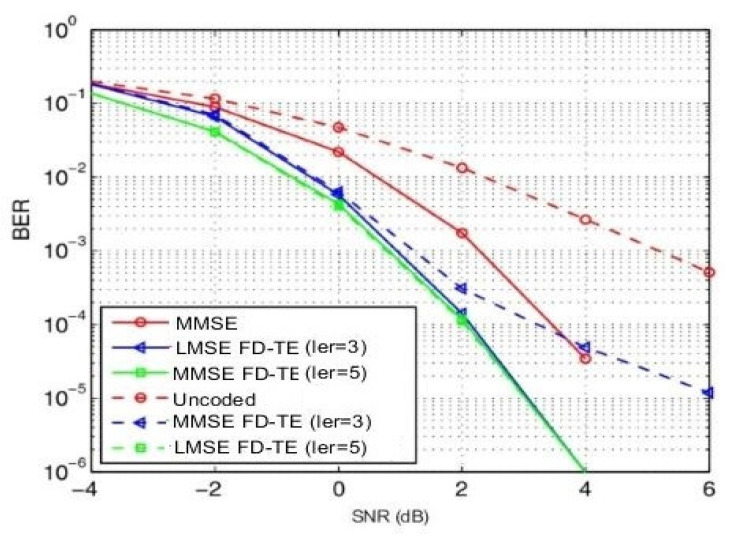
BER performance comparison of the proposed MMSE-FDTE and LMSE equalization algorithms of uplink NB-IoT systems using QPSK with different numbers of iterations.

In Figure 6 and Figure 7, we present the BER performance of the decoder with CRC error detection and effect of coding rates (R = 1/2 and R = 1/3), respectively. Figure 8 and Figure 9 show the BER performance of our proposed receiver for $\Delta f_{n}=15\;\mathrm{kHz}$ and QPSK constellation using the proposed MMSE FDTE compared to other LMSE equalizer algorithms. At the first decoding iteration, the proposed FDTE yielded significant performance gains of about 3.2 dB and 3.5 dB, which tended to converge after four decoding iterations. Similarly, Figure 7 illustrates the BER performance of our proposed receiver for $\Delta f_{n}=3.75$ using the QPSK constellation scheme. In both Figure 6 and Figure 7, the proposed receiver has slight improvement when working with a lower order modulation scheme over the conventional FDTE algorithm for both $\Delta f_{n}=15\;\mathrm{kHz}$ and $\Delta f_{n}=3.75\;\mathrm{kHz}\;\mathrm{SCS}$, while yielding a significant performance gain over the conventional LMSE FDTE in [29], which is considered enough for desired uplink single tone transmission.

### 3.3. Complexity Analysis

Computation complexity is derived from equalizer vectors. Symbol estimates in terms of complex multiplications and additions as summarized in Table 2. The conventional FD-MMSE equalization algorithm as given by (19) requires *M* complex multiplications to estimate the equalizer symbols. In the proposed FD-MMSE equalizer scheme, to suppress the MMSE estimation error, additional operations are required to be configured including the K-point IFFT operation and nK-point FFT operation. Hence, its computational complexity in terms of cost is higher in comparison with conventional MMSE and LMSE schemes [21]. As a result, the additional computational cost consists of $K\left[n\log_{2}\left(nK\right)+\log_{2}\left(K\right)\right]$ complex additions and $\left(K/2\right)\left[n\log_{2}\left(nK\right)+\log_{2}\left(K\right)\right]$ complex multiplications.

The complexity of the LMSE equalizer is approximately similar to the DFT-based scheme in [20]. Besides, the performance of the proposed FD-MMSE turbo equalization operating at a low SNR region (i.e., SNR <0 dB) is better than the corresponding turbo equalization proposed in [22] in uplink NB-IoT systems.

## 4. Conclusions and Future Work

In this work, an analytic LTE-based uplink NB-IoT baseband model is derived. We proposed a low complexity receiver framework based on the MAP turbo decoding scheme and FDTE equalizer algorithms. Furthermore, the analysis and mathematical formulation of our proposed scheme are presented, including the detection sequence analyzed using one-tap equalization and frequency domain operations. Simulation of BER performance shows that our scheme is fairly robust over conventional MMSE FDTE, which can enhance transmission reliability of UE data in NPUSCH and hence reduce computation complexity suitable for LPWA NB-IoT systems.

Future study will look into the modeling of the NB-IoT network PHY layer, the proposals of energy efficiency technology for MCS selection, the improvement of data rate and the management of network scalability, which can be a framework based on the modified MAP decoding and MMSE equalization to improve the transmission reliability of uplink user data and achieve wide-area coverage.

The proposed schemes can also be extended to perform NDMRS channel estimation for carrier frequency offset (CFO) compensation to estimate the frequency error with self-interference cancellation (SIC) on the received NPUSCH information sequence, while canceling interference at the receiver will also be a topic of interest. Additionally, this algorithm can also be extended to HARQ for the NPUSCH uplink control channel as well as being energy-efficient to optimize the ACK/NACK feedback signaling. In this framework, signaling only needs to indicate through the DCI, which are lost during the uplink transmission, and hence allows saving power consumption of the NB-IoT network.

## Figures and Tables

**Table 1:** Technical specification of LPWA network technology.

Technology	LTE-A	EC-GSM	NB-IoT
Deployment	In-band LTE	In-band GSM	In-band, Guard-band, and Stand-alone
Bandwidth	1.08 MHz	200 kHz per channel.	180 kHz
Network Coverage	155.7 dB	164 dB, with 33 dBm power class. 154 dBm, with 23 dBm power class	164 dB for stand-alone and FFS for others
Downlink Technology	OFDMA, with 15 kHz SCS	TDMA, FDMA, GMSK and 8 PSK	OFDM with 15 kHz SCS
Uplink Technology	SC-FDMA with 15 kHz SCS	TDMA, FDMA, GMSK and 8 PSK	Single-tone SC-FDMA with both 15 kHz and 3.75 kHz. Multi-tone SC-FDMA with 15 kHz
Data Rates	1 Mbps for both UL and DL	70 Kbps with TDMA, FDMA for both UL and DL and 240 kbps with 8 PSK	28 kbps for DL and 63 kbps for UL
Duplexing	FD and HD (type B), FDD	HD and FDD	HD (type B) and FDD
Power saving	PSM, ext. 1 DRX, C-DRX	PSM, ext. 1-DRX	PSM, ext. 1 DRX, C-DRX
Power class	23 dBm, 20 dBm	33 dBm, 23 dBm	23 dBm, other TBD

**Table 2:** Complexity comparison.

Operation	Conv. MMSE [21]	Conv. LMSE [22]	Proposed FD-MMSE
Complex Addition	$K\left[\log_{2}\left(K\right)+2\right]$	2K(K−1)	$K\left[n\log_{2}\left(nK\right)+\log_{2}\left(K\right)\right]$
Complex multiplication	$K\left[\left(1/2\right)\log_{2}\left(K\right)+2\right]$	$K^{3}+2K^{2}$	$K\left[1+\left(1/2\right)\left[n\log_{2}\left(nK\right)+\log_{2}\left(K\right)\right]\right]$
Complexity	Medium complexity	High Complexity	Medium complexity

## Data Availability

Not Applicable.
